# PSII supercomplex disassembly is not needed for the induction of energy quenching (qE)

**DOI:** 10.1007/s11120-022-00907-w

**Published:** 2022-03-18

**Authors:** Ludwik W. Bielczynski, Pengqi Xu, Roberta Croce

**Affiliations:** Biophysics of Photosynthesis, Department of Physics and Astronomy, Faculty of Science, Vrije Universiteit Amsterdam, Amsterdam, the Netherlands

**Keywords:** Non-photochemical quenching, Photosystem II, Light-harvesting complexes, Xanhophylls

## Abstract

Photoprotection by non-photochemical quenching is important for optimal growth and development, especially during dynamic changes of the light intensity. The main component responsible for energy dissipation is called qE. It has been proposed that qE involves the reorganization of the photosynthetic complexes and especially of Photosystem II. However, despite a number of studies, there are still contradictory results concerning the structural changes in PSII during qE induction. The main limitation in addressing this point is the very fast nature of the off switch of qE, since the illumination is usually performed *in folio* and the preparation of the thylakoids requires a dark period. To avoid qE relaxation during thylakoid isolation, in this work quenching was induced directly on isolated and functional thylakoids that were then solubilized in the light. The analysis of the quenched thylakoids in native gel showed only a small decrease in the large PSII supercomplexes (C_2_S_2_M_2_/C_2_S_2_M) which is most likely due to photoinhibition/light acclimation since it does not recover in the dark. This result indicates that qE rise is not accompanied by a structural disassembly of the PSII supercomplexes.

**Supplementary Information:**

The online version contains supplementary material available at 10.1007/s11120-022-00907-w.

## Introduction

Light is essential for photosynthesis, but when absorbed in excess, it can damage the photosynthetic apparatus. To avoid this, photosynthetic organisms have evolved several photoprotective mechanisms that permit them to respond to changes in light conditions (Ruban et al. 2012; Bassi and Dall’Osto 2021).

Photoprotection by non-photochemical quenching (NPQ) is important for growth and development, especially during dynamic changes of light intensity (Frenkel et al. 2009). In general, NPQ is a very broad term, which includes state transitions [qT; (Bellafiore et al. 2005; Pesaresi et al. 2009)], photoinhibition [qI; (Quick and Stitt 1989)], zeaxanthin-dependent quenching [qZ; (Jahns and Holzwarth 2012)], and LCNP-dependent quenching [qH; (Malnoe et al. 2018)]. However, in most physiological conditions, the main NPQ component is qE, a process in which the energy absorbed in excess is dissipated as heat (Ruban 2016). The process is triggered by the low luminal pH that activates the PsbS protein (Li et al. 2000) and the xanthophyll cycle (Demmig-Adams (Demmig-Adams 1990).

Under high light, the xanthophyll Violaxanthin is transformed to Zeaxanthin via Antheraxanthin by the enzyme violaxanthin de-epoxidase as reviewed in Jahns and Holzwarth (2012). The reverse reaction is catalyzed by the zeaxanthin epoxidase. Although the exact role of Zeaxanthin in NPQ is not yet fully understood (Johnson et al. 2009; Xu et al. 2015), it is clear that this xanthophyll is essential to reach the maximum level of NPQ.

During NPQ, excitation energy quenching occurs in Photosystem II (PSII). PSII-LHCII is a water-plastoquinone oxido-reductase composed of many pigment-binding proteins and which can be functionally divided into two moieties: the core, which contains the reaction center (RC), where charge separation occurs, and several light-harvesting complexes (LHCs), which enhance the capacity of the core to harvest light. In vascular plants, the core is mostly found in dimeric form (C_2_), in which each monomer binds several LHCII trimers (LHCII-S, LHCII-M, or LHCII-L bound with strong and moderate affinity, or loosely, respectively) and one each of the minor antennae CP24, CP26, and CP29 (Croce and van Amerongen 2020).

Due to the complicated nature of the NPQ, it is not yet clear if this process involves any structural changes in the PSII. Some data suggest that NPQ involves the association and disassociation of antennae from the core (Holzwarth et al. 2009; Betterle et al. 2009; Johnson et al. 2011), while other experiments indicate that the antenna size of PSII even increases during NPQ (Belgio et al. 2014). In our previous work (Bielczynski et al. 2016), we observed that during short HL illumination (0.5–6 h) the changes in the PSII supercomplexes were small and occurred mainly in the large PSII supercomplexes (C_2_S_2_M_2_ and C_2_S_2_M). However, the illumination was performed *in folio* and the fast qE component was very likely relaxing during thylakoid isolation. As a result, in those experiments we could not monitor the changes due to the fast qE component (PsbS-dependent), but only those related to the presence of Zeaxanthin, which survives the biochemical preparation.

In this work, to avoid qE relaxation, we have induced quenching directly on isolated and functional thylakoids, and solubilized them in the light. The results indicate that PSII disassembly is not essential for qE.

## Materials and methods

### Plant material

*Arabidopsis thaliana* (ecotype Col-0) WT seeds were sown on MS agar plates. After 5–7 days the seedlings were transplanted to final pots. Plants were grown for 7 weeks in growing chambers (AR-36L, Plant Climatics Percival) at 70% RH, 21 °C, in a photoperiod of 8/16 h (day/night) and under 200 $\mu$mol of photons ${\text{m}}^{-2}$ ${\text{s}}^{-1}$.

### Thylakoid isolation and solubilization

The thylakoid isolation was performed as previously described in Gilmore et al. (1998) in three biological repetitions (meaning on plants grown at different times). Plants were dark-adapted over night before the treatment. Leaves were collected and kept for a short time in an ice-water bath before thylakoid isolation. Around 10 leaves from 1 to 3 plants were used for each preparation. Plants thylakoids were immediately used after preparation. The same thylakoids were subjected to three different treatments: (1) dark, the membranes were maintained and solubilized in darkness; (2) NPQ, the membranes were illuminated for 10 min to reach the maximum of NPQ and solubilized under light with stirring; and (3) recovery, the membranes were solubilized after 10-min illumination and 20 min at RT. Solubilization was performed with 0.6% alpha-DDM final concentration at room temperature, under stirring and the samples were transferred directly to ice.

### Fluorescence measurements

The fluorescence measurements were performed using DualPAM-100 (Walz). The measuring light (ML), actinic light (AL), and saturating pulse (SP) were of intensities around 5, 531, and 2000 $\mu$mol of photons ${\text{m}}^{-2}$ ${\text{s}}^{-1}$, respectively. The SP was 500 ms long. A fixed amount of isolated thylakoid membranes, corresponding to 500 $\mu$g of Chls, in 0.5 ml volume, were supplemented with methyl viologen (MV) and sodium ascorbate (NaAsc), to a final concentration of 50 $\mu$ M MV and 30 mM NaAsc. The samples during the measurements were stirred and subjected to a SP to estimate the maximal fluorescence from dark-adapted sample ${F}_{\text{M}}$), after a prior ${F}_{0}$ (minimal fluorescence from dark-adapted samples) measurement. Right after the SP, AL was switched on, and over 10 min of illumination each 2 min a SP was used to measure the maximal fluorescence from light-adapted samples) (Baker 2008). Afterward, to probe the recovery phase, the light was switched off and six SPs were triggered over 20 min (intervals: 30 s, 30 s, 1 min, 2 min, 8 min, and 8 min).

### Pigment isolation

The Chl *a*/*b* ratio and Chl/Car ratio were determined from absorption spectra of 80% acetone extracts measured with a Carry 4000 spectrophotometer (Varian). The absorption spectra were fitted with the spectra of individual pigments in the same solvent, as previously described (Croce et al. 2002). The quantification of different carotenoids was performed by HPLC using a System Gold 126 Solvent module and 168 Detector (Beckman Coulter) as previously described in (Gilmore and Yamamoto 1991) with modifications (Xu et al. 2015).

### BN-PAGE and 2D-PAGE

BN-PAGE and 2D-PAGE were performed as previously described in (Jarvi et al. 2011) with modifications from (Bielczynski et al. 2016). For BN-PAGE, we used resolving gels with a 4–12.5% acrylamide gradient. The BN-PAGE was documented using a standard transmission scanner. To obtain the integrated optical density (IOD) profiles, in each lane, we summed the optical densities (pixel intensities in the blue channel of the RGB picture) across the resolving axis.

## Results

As our goal was to determine the changes in the organization of PSII induced by qE, which relaxes very fast in the dark, we induced NPQ directly on isolated, functional thylakoids, and we solubilized the membranes under light. The solubilized material was then analyzed by BN-PAGE.

**Fig. 1:**
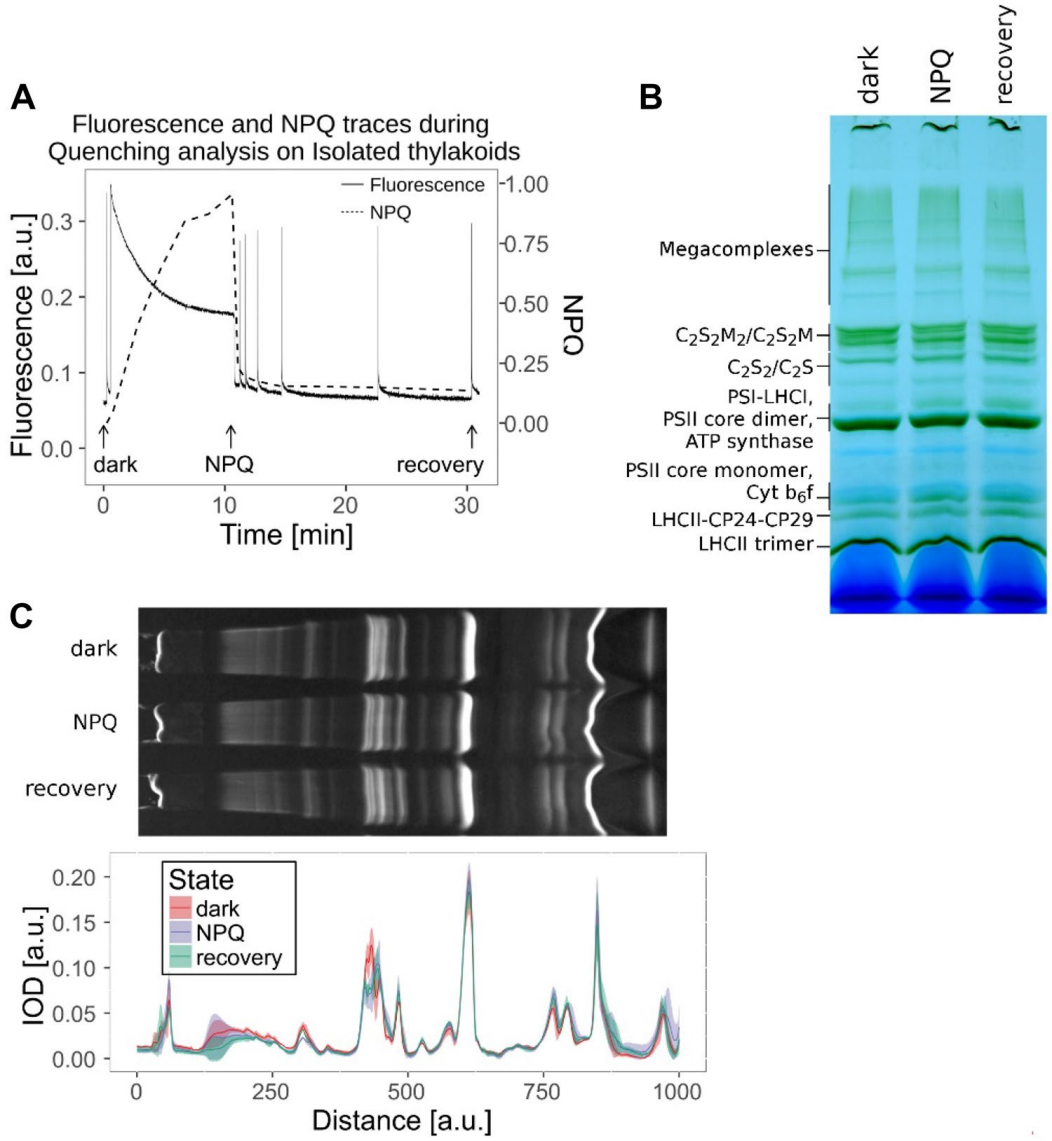
NPQ induction on functional thylakoids and analysis of the photosynthetic complexes. **A** Chlorophyll fluorescence and NPQ traces (solid and dashed lines, respectively) of isolated and functioning thylakoids of *A*. *thaliana*. The arrows indicate the time-points at which the thylakoids were solubilized: dark, NPQ, and recovery. **B** BN-PAGE of the samples solubilized at the time-points shown in panel A. **C** Analysis of the BN-PAGE. Top panel: Chlorophyll containing bands (blue channel of an RGB picture of a BN-PAGE) of the thylakoid membranes and solubilized at the time-points shown in panel (**A**). Bottom panel: Integrated Optical Density (IOD) profiles of the BN-PAGE lanes shown in panel (**B**) and (**C**): dark, NPQ, and recovery (red, violet, and green trace, respectively). The shadows are the standard deviation from two biological replicas, each with 2–3 independent repetitions (*n* = 5)

First, we ensured that the isolated thylakoids were functional. After isolation, we performed a standard quenching analysis on the thylakoids using an exogenous electron acceptor, methyl viologen (MV). As shown in Fig. 1A, at the end of 10-min illumination, we reached an NPQ value of around 1 (light samples). The kinetics of the NPQ induction was slightly slower when compared to the analogous measurements performed *in folio*, on plants grown under the same conditions (Bielczynski et al., 2016). However, qE was the major component of NPQ, as after switching off light, NPQ dropped fast (within 2 min) by more than 80%. To determine the organization of the PSII complexes at time zero, in the absence of NPQ, an aliquot of isolated thylakoid membranes was kept (and solubilized) in darkness (dark/unquenched sample). To exclude contributions from other NPQ components, we also analyzed the membranes after recovery: an aliquot of thylakoids was solubilized after illumination and an additional 20 min of darkness, a condition in which qE is relaxed and the remaining quenching is qI/ qZ (recovery sample).

As NPQ also depends on the xanthophyll cycle, we performed a pigment analysis to monitor the epoxidation state of the xanthophylls (Table 1). Similar to what reported before (Xu et al. 2015), during the 10 min of illumination, around 40% of the Violaxanthin pool was de-epoxidized to Antheraxanthin and Zeaxanthin. During the recovery phase, the de-epoxidation level did not revert to the initial dark level, in part probably because the enzyme needed for the re-epoxidation of Antheraxanthin and Zeaxanthin was lost during isolation, as it is located in the chloroplast stroma (Siefermann and Yamamoto 1975). As expected no differences in the chlorophyll content were observed between the three samples.

**Table 1:** Pigment analysis of functional, thylakoids solubilized in dark, NPQ, and recovery state

	Dark	NPQ	Recovery
Chl a/b ratio	3.23 ± 0.05	3.21 ± 0.05	3.22 ± 0.05
Chl/Car ratio	3.57 ± 0.01	3.55 ± 0.03	3.54 ± 0.07
Lut	13.05 ± 0.09	13.15 ± 0.13	13.22 ± 0.09
β-Car	7.92 ± 0.07	7.67 ± 0.13	7.77 ± 0.09
Neo	3.87 ± 0.02	3.78 ± 0.06	3.78 ± 0.07
Vio	3.21 ± 0.03	1.95 ± 0.01	1.98 ± 0.02
Ant	N.D	0.20 ± 0.00	0.20 ± 0.00
Zea	N.D	1.37 ± 0.10	1.33 ± 0.00
(0.5*Ant + Zea)/(Vio + Ant + Zea)	N.D	0.42 ± 0.02	0.41 ± 0.00

The chlorophyll a/b (Chl a/b) ratio and chlorophyll/carotenoid (Chl/Car) ratio were determined by fitting the absorption spectra of the 80% acetone extracts from isolated thylakoid membranes. The same extracts were used for the carotenoids’ quantification by HPLC: neoxanthin (Neo), violaxanthin (Vio), lutein (Lut), and β-carotene (β-car). All carotenoids were calculated per 100 Chls (*n* = 3)

**Fig. 2:**
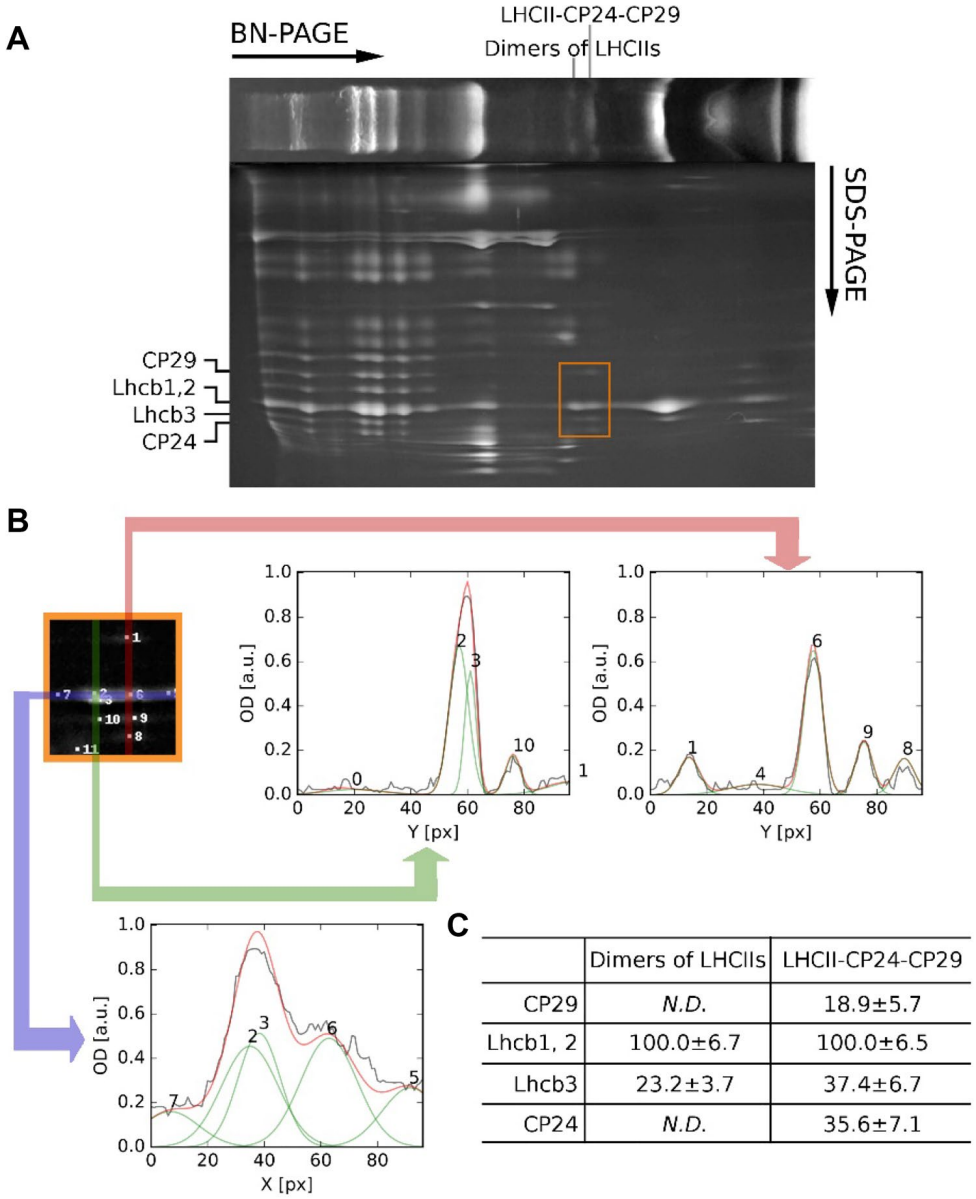
Dimers of LHCII trimers in BN-PAGE. **A** At the top, an example of BN-PAGE strip is shown. The proteins composing the photosynthetic complexes were separated on the 2D-PAGE and stained with Coomassie blue. The rectangular orange selection represents the region where dimers of LHCIIs and LHCII-CP24-CP29 are closely migrating and which is reproduced enlarged in (**B**). **B** Example of the data analysis. The close up of the region of interest with the location of the fitted 2D Gaussians is shown in the orange frame. The IOD profile where complexes are overlapping (purple arrow), and the composition of the dimers of LHCIIs and LHCII-CP24-CP29 (green and red arrow, respectively) is shown. The original and fitted traces are plotted (black and red color, respectively). Gaussians composing of the fitted trace are numbered and plotted as green traces. **C** In the table, the amount of CP29, Lhcb3, and CP24 in dimers LHCIIs and LHCII-CP24-CP29 normalized to Lhcb1, Lhcb2 are presented. The data are the result of six repetitions (*n* = 6)

We then proceeded to investigate the organization of PSII supercomplexes by BN-PAGE in the three samples: dark, NPQ, and recovery (Fig. 1). For the initial quantification of the PSII supercomplexes, we looked at the integrated optical density (IOD) profiles from the BN-PAGE (Fig. 1C). We used only the blue channel of the RGB picture. Since the Coomassie stain does not absorb in this spectral region, this enables us to obtain a clean signal coming from the Soret band of the Chls (Supplemental Fig. 1). However, as in this region carotenoids absorb as well, we might get an error due to the different pigment composition of the complexes. To check if this is indeed the case, we compared the results obtained with this method with those obtained from the precise quantification of the proteins based on the estimate of the Lhcb1,2 protein band resolved using 2D-PAGE (Bielczynski et al. 2016). This led to similar results (Supplemental Fig. 2), indicating that the analysis of the blue channel represents a suitable method to quantify photosynthetic complexes directly from a BN-PAGE. Besides the previously described chlorophyll-binding fractions containing the supercomplexes and subcomplexes of PSI and PSII (Jarvi et al. 2011; Bielczynski et al. 2016), we observed an additional green band with a MW slightly higher than that of the LHCII-CP24-CP29 complex (Fig. 1). To determine the protein content of this band, we ran a 2D-PAGE (Fig. 2A). As this band migrated very close to the LHCII-CP24-CP29 band (Fig. 2B), we performed a 2D Gaussian fit of the region of interest. The additional band was composed only of Lhcb1, Lhcb2, and Lhcb3 (Fig. 2C), and showed a lower relative amount of Lhcb3 when compared to the LHCII-CP24-CP29 complex. According to its MW and composition, we can conclude that this band contains a dimer of LHCII trimers.

When separating the complexes from the thylakoid membranes on the BN-PAGE, we also observed bands with MW higher than the PSII supercomplexes, described previously as PSII megacomplexes (Jarvi et al. 2011). Their amount decreased in both the NPQ and recovery samples as compared to the dark sample, probably due to their low stability induced by high light after solubilization of the membrane.

Next, we quantified all the other PSII- and LHCII-containing fractions (Table 2), by integrating the IOD for each fraction. As the C_2_S_2_M_2_ and C_2_S_2_M, and C_2_S_2_ and C_2_S PSII fractions often overlapped, we integrated the signal of the two larger complexes (C_2_S_2_M_2_ and C_2_S_2_M) and of the two smaller (C_2_S_2_ and C_2_S) together and in the following we call these two fractions “large” and “small” PSIIs. To correct for the differences in loading and solubilization of the samples, we normalized each fraction to the total IOD of the gel lane. One of the disadvantages of quantifying the complexes straight from the BN-PAGE is that we cannot follow the changes in the amount of the dimeric core since its MW is similar to that of the highly abundant PSI-LHCI supercomplex and in consequence, their contributions cannot be disentangled (Fig. 2). However, in our sample, the amount of monomeric core is below the detection limit, and in the thylakoid membrane, the amount of dimeric core is normally very low since most of the PSII is present in the form of supercomplexes. We thus assume that the amount of PSII core is negligible. This is supported by the small (and statistically not significant) difference in the band containing PSI-LHCI + PSII core in the three conditions.

**Table 2:** Quantification of PSII- and LHCII-related fractions in dark, NPQ, and recovery state

Complex	Condition		
	Dark	NPQ	Recovery
C_2_S_2_M_2_/C_2_S_2_M	35.9 ± 3.7^a^	30.0 ± 3.8^b^	30.6 ± 3.0^b^
C_2_S_2_/C_2_S	10.7 ± 1.4	12.3 ± 2.1	12.7 ± 1.6
Dimers of LHCIIs	11.3 ± 2.1	12.8 ± 0.8	12.9 ± 1.6
LHCII-CP24-CP29	9.7 ± 0.9	10.3 ± 0.7	9.2 ± 0.8
LHCII trimers	24.9 ± 3.7	27.1 ± 3.0	22.7 ± 3.6
LHCII monomers	7.4 ± 1.5	7.4 ± 2.3	7.4 ± 2.0

Specific PSII complexes from the IOD profiles of the BN-PAGE shown in Fig. 1A are presented: Large supercomplexes (C_2_S_2_M_2_/(C_2_S_2_M), Small PSII supercomplexes (C_2_S_2_/(C_2_S), dimers of LHCII trimers, LHCII-CP24-CP29, LHCII trimers, and LHCII monomers. They were quantified in the thylakoids solubilized in dark, NPQ, and recovery state. The values are presented as percentage of the total amount of all fractions. Two groups of means from a pairwise Tukey HSD test are labeled with indexes with letters (*p* value < 0.1). The statistics were calculated from two biological replicas, each consisting of 2–3 independent repetitions (*n* = 5)

Compared to the dark sample, after illumination, we observed only a small decrease in the large PSIIs (around 6%). We also observe a small increase (insignificant statistically) in the amount of all the other fractions (besides LHCII monomers), which taken together, become statistically significant. We can conclude that the components of the large PSIIs when disassembled are distributed between the other PSII and LHCII fractions.

Finally, it is important to note that the amount of the large PSIIs did not increase in the recovery sample while qE was relaxed. This suggests that the small changes that we observed between dark and NPQ conditions (*i.e.*, disassociation of PSII supercomplexes, increase in the dimers of LHCII, LHCII-CP24-CP29, and LHCII trimers) could not be attributed to the reorganization of the membrane during qE. We conclude that it is probably the result of photoinhibition/photodamage or the beginning of the long-term acclimation.

## Discussion

The results described above show that the rearrangement of the PSII supercomplexes C_2_S_2_ and C_2_S_2_M_2_ is not a major factor for qE, since the difference in the distribution of the proteins between PSII subcomplexes and supercomplexes in dark and light samples was very small. Moreover, this difference remained unaltered in the recovery sample when the qE component had relaxed, suggesting that the rearrangement resulted from photoinhibition or high light-induced long-term disassembly. Surprisingly, the amount of LHCII-CP24-CP29 complex did not change upon light treatment. This is at variance with previous results that showed that the LHCII-CP24-CP29 complex disassembled during HL illumination (Betterle et al. 2009). This disassembly was shown to be PsbS-dependent and thus suggested to be related to qE. A possible explanation for this difference is the length of the HL treatment: 30 min on intact plants vs 10 min on functional thylakoids. It is thus possible that the disassembly of the LHCII-CP24-CP29 subcomplex is part of a long-term acclimation strategy, which also involves PsbS. This seems to be supported by the fact that the HL treatment on the intact plant was followed by the purification of thylakoids and by their solubilization. Those procedures include long dark periods, during which qE relaxes.

Reduction of the PSII size during NPQ was also observed by freeze-fracture electron microscopy (Johnson et al. 2011). Since our results show that C_2_S_2_M_2_ and C_2_S_2_ complexes are not affected during NPQ, the changes observed by Johnson et al. are most likely due to the physical disconnection of other complexes that belong to a different pool of LHCII’s: In plants there are more LHCII trimers than those present in the C_2_S_2_M_2_ supercomplex. The association of these extra trimers to PSII is loose and does not survive purification, but in the membrane these trimers act as a functional PSII antenna (Croce 2020) and in normal light conditions they are located in between the PSII supercomplexes (Kouril et al. 2013). The freeze-fracture data of Johnson and colleagues (Johnson et al. 2011) also show that the distance between PSII supercomplexes is reduced in NPQ conditions, thus suggesting that these trimers are removed from the space between the photosystems and are thus likely the ones clustering in other parts of the membrane. The extra LHCII trimers are thus likely to form the main component of the band containing dimers of LHCII trimers visible in our blue native gel.

In conclusion, our results show that at least a large part of the C_2_S_2_ and C_2_S_2_M_2_ supercomplexes are still intact in NPQ conditions, indicating that the disconnection of the strongly and moderately bound LHCII trimers from the PSII core is not a requisite for NPQ.

## Supplementary Information

Below is the link to the electronic supplementary material.

Supplementary file1 (DOCX 554 KB)
