# Supplementary material for: PSII supercomplex disassembly is not needed for the induction of energy quenching (qE)
Source: Photosynth Res. 2022 Mar 18;152(3):275–81. doi: 10.1007/s11120-022-00907-w (PMC9458576; doi:10.1007/s11120-022-00907-w)
Supplement: Supplementary file 1 — Supplementary file1 (DOCX 554 KB) [file 11120_2022_907_MOESM1_ESM.docx]

**Supplementary figures**


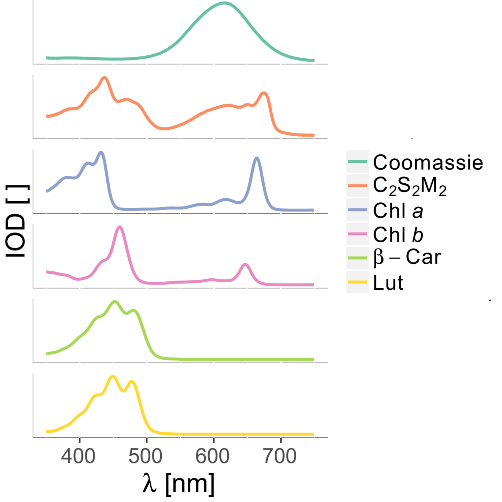


***Supplemental Figure 1. Absorption spectra profiles.*** *Comparison of the absorption profiles of Coomassie and C 2 S 2 M 2 PSII supercomplexes in buffer, and Chl a and b, β-Car and Lut in 80% of acetone was shown.*


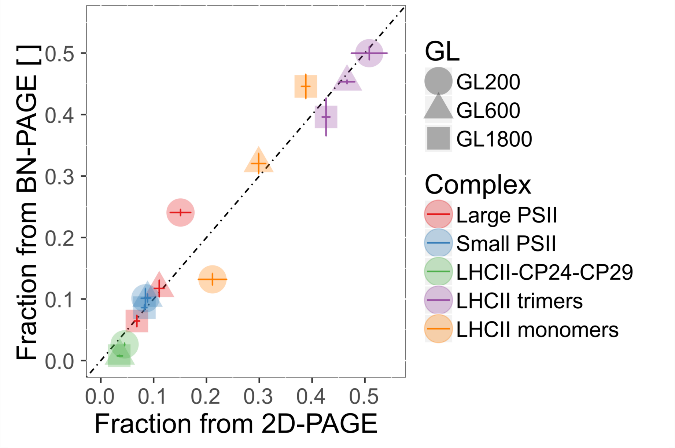


***Supplemental Figure 2. Comparison of BN-PAGE and 2D-PAGE based quantification of Lhcb1 and 2 proteins.*** *Large and small PSIIs, LHCII-CP24-CP29, LHCII trimers and monomers (red, blue, green, violet and orange, respective) from plants grown under 200, 600 and 1800 µmol of photons m^−2^ s^−1^ (GL200, 600 and 1800; circles, triangles and rectangles, respectively) were quantified with two methods: IOD profile of the BN-PAGE (the blue channel), and IOD profile of the Lhcb1 and Lhcb2 proteins in the 2D-PAGE (from Bielczynski et al., 2016).*
